# Mathematical prediction with pretreatment growth rate of metastatic cancer on outcomes: implications for the characterization of oligometastatic disease

**DOI:** 10.3389/fonc.2023.1061881

**Published:** 2023-05-29

**Authors:** Yerim Shin, Jee Suk Chang, Yeseul Kim, Sang Joon Shin, Jina Kim, Tae Hyung Kim, Mitchell Liu, Robert Olson, Jin Sung Kim, Wonmo Sung

**Affiliations:** ^1^ Department of Biomedical Engineering and of Biomedicine & Health Sciences, College of Medicine, The Catholic University of Korea, Seoul, Republic of Korea; ^2^ Department of Radiation Oncology, Yonsei University College of Medicine, Seoul, Republic of Korea; ^3^ BC Cancer - Vancouver Centre, Vancouver, BC, Canada; ^4^ Division of Medical Oncology, Department of Internal Medicine, Yonsei University College of Medicine, Seoul, Republic of Korea; ^5^ Department of Radiation Oncology, Nowon Eulji Medical Center, Eulji University School of Medicine, Seoul, Republic of Korea; ^6^ BC Cancer, Centre for the North, Prince George, BC, Canada; ^7^ Department of Radiation Oncology, Yonsei Cancer Center, Heavy Ion Therapy Research Institute, Yonsei University College of Medicine, Seoul, Republic of Korea

**Keywords:** immune checkpoint inhabitor, melanoma, mathematical modeling, oligometastasis, tumor growth rate

## Abstract

**Background:**

Oligometastatic disease (OMD) represents an indolent cancer status characterized by slow tumor growth and limited metastatic potential. The use of local therapy in the management of the condition continues to rise. This study aimed to investigate the advantage of pretreatment tumor growth rate in addition to baseline disease burden in characterizing OMDs, generally defined by the presence of ≤ 5 metastatic lesions.

**Methods:**

The study included patients with metastatic melanoma treated with pembrolizumab. Gross tumor volume of all metastases was contoured on imaging before (TP_-1_) and at the initiation of pembrolizumab (TP_0_). Pretreatment tumor growth rate was calculated by an exponential ordinary differential equation model using the sum of tumor volumes at TP_-1_ and TP_0_ and the time interval between TP_-1_. and TP_0_. Patients were divided into interquartile groups based on pretreatment growth rate. Overall survival, progression-free survival, and subsequent progression-free survival were the study outcomes.

**Results:**

At baseline, median cumulative volume and number of metastases were 28.4 cc (range, 0.4-1194.8 cc) and 7 (range, 1-73), respectively. The median interval between TP_-1_ and TP_0_ was -90 days and pretreatment tumor growth rate (×10^-2^ days^-1^) was median 4.71 (range -0.62 to 44.1). The slow-paced group (pretreatment tumor growth rate ≤ 7.6 ×10^-2^ days^-1^, the upper quartile) had a significantly higher overall survival rate, progression-free survival, and subsequent progression-free survival compared to those of the fast-paced group (pretreatment tumor growth rate > 7.6 ×10^-2^ days^-1^). Notably, these differences were prominent in the subgroup with >5 metastases.

**Conclusion:**

Pretreatment tumor growth rate is a novel prognostic metric associated with overall survival, progression-free survival, and subsequent progression-free survival among metastatic melanoma patients, especially patients with >5 metastases. Future prospective studies should validate the advantage of disease growth rate plus disease burden in better defining OMDs.

## 1 Introduction

Metastatic cancer has long been considered an incurable disease (1). However, in 1995, Hellman and Weichselbaum first proposed the oligometastatic disease (OMD) concept, suggesting that biologically limited small number of bundles or slowly progressing metastatic cancers could be curable (2). In addition, studies on ablation of gross metastatic tumors using stereotactic ablative radiotherapy (SABR) or surgery in patients with OMD from various histology has shown an improvement in survival outcomes; however, phase III trials remain pending in these studies (3, 4).

Since there are no biomarkers to define OMD, clinical trials have used either five or three metastatic lesions on imaging as the cutoff (5). Previous trials involving 1–3 or 1–5 metastases (6) have reported a sizable risk of rapid or widespread metastatic progression following SABR as being characteristic of OMD, thus raising a valid concern on the clinical application of SABR for OMD treatment in real-world practice. Pretreatment baseline tumor burden is widely acknowledged as an important indicator for cancer therapy and helps distinguish OMDs from polymetastatic diseases (7, 8).In the same context, randomized clinical trials testing new drugs against cancer used the baseline tumor burden as a stratification factor (7).

The present study was designed to determine whether OMD can be clinically defined based on the pretreatment growth rate of metastatic tumors. Using a cohort of metastatic melanoma patients treated with an immune checkpoint inhibitor (ICB), we developed a mathematical model with a simple exponential ordinary differential equation (ODE) to determine tumor growth rate of total disease burden prior to ICB administration, and thereafter, determine the effect of tumor growth rate on patient outcomes. Advancements in the methodological assessment of tumor growth may serve as a prognostic tool for per-patient response to cancer therapy.

## 2 Meterials and methods

### 2.1 Study design

**Figure 1:**
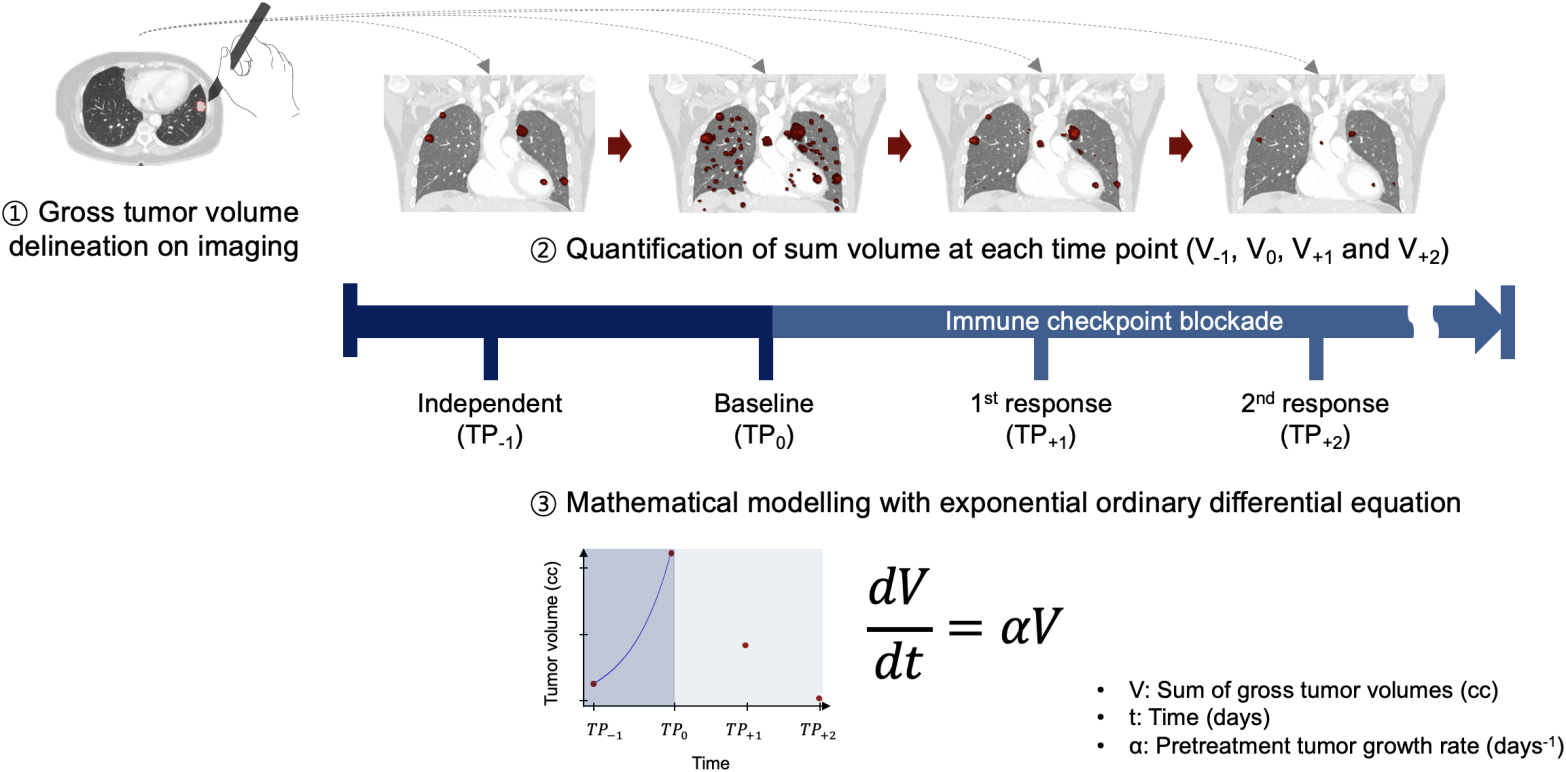
Schematic overview of study design. TP, time point.

This study was approved by the Institutional Review Board (4-2021-1683) of Severance Hospital, Seoul, Republic of Korea, and the requirement for patient informed consent was waived because of the study’s retrospective design. We identified 86 patients with metastatic malignant melanoma who were treated with pembrolizumab monotherapy at a single center between 2015 and 2020. The patient’s demographic information, clinical and tumor characteristics, time interval between the time of ICB treatment start (baseline) and pretreatment time point (TP**_-1_**), response to ICB treatment, and follow-up status were from a database (**Figure 1**). Patient data and tumor characteristics are summarized in **Table 1**. A separate study was conducted using the same study cohort to compare the prognostic value of a cumulative 3-dimensional (3D) volume of all metastases and counting the total number of metastases as a surrogate for the disease burden (8).

**Table 1:** Patient characteristics.

Variables	Number of patients	%
Sex		
Male	47	54.7%
Female	39	45.3%
Age		
Median (IQR)	57(49-65)	
BRAF mutation		
No	58	67.4%
Yes	11	12.8%
Unknown	17	19.8%
Treatment line		
First line	62	72.1%
≥2^nd^ line	24	27.9%
Primary type		
Mucosal	20	23.3%
Acral	13	15.1%
Non-CSD	37	43.0%
CSD	2	2.3%
Others	14	16.3%
Metastasis site		
Brain involvement	11	12.8%
Liver involvement	23	26.7%
Bone involvement	21	24.4%
Lung involvement	32	37.2%
Lymph node involvement	46	53.5%
Others	18	20.9%

CSD, chronic sun damage.

### 2.2 Study cohort

In this study cohort, patients with new tumor recurrence or metastatic tumor after prior treatment were treated with pembrolizumab, an anti-programmed cell death-1 antibody, monotherapy. Basic administration protocol was intravenous injection of 2 mg/kg doses every 2 or 3 weeks. Drug administration continued until disease progression or the 36^th^ cycle, whichever came first. Baseline workup consisted of pathologic confirmation and computed tomography (CT) imaging of the chest and abdomen-pelvic area as well as the neck or upper/lower extremities, if necessary. When CT images showed equivocal findings, magnetic resonance imaging (MRI) was performed. In addition, brain MRIs to evaluate intracranial disease were performed when patients complained of neurologic symptoms or at physicians’ discretion if the patient was asymptomatic. Positron emission tomography-computed tomography (PET-CT) scan was performed in most patients to evaluate the extent and severity of any systemic disease. In brain metastasis, stereotactic radiosurgery and/or external beam radiotherapy (RT) was administered after a multidisciplinary discussion for intracranial diseases. Patients presenting with bleeding, pain, or mass effect were administered a local palliative RT to treat the symptom-causing lesion. Regular follow-up involving CT imaging of the chest, abdomen-pelvic area, and other areas, if necessary, were performed every three cycles of pembrolizumab administration.

### 2.3 Volumetric assessment

For quantitative assessment, board-certified radiation oncologists delineated the gross tumor volume of all metastatic lesions that appeared on available CT, PET-CT, and/or MRI (a) at the time of pembrolizumab initiation (baseline, TP_0_) and (b) independent from any clinical intervention (TP_-1_) using the MIM contouring tool (MIM Software Inc., version 7.1.7, Cleveland, OH, USA). Axial cut images with slice thickness ≤ 3 mm were used for tumor delineation, and multi-modality fusion was performed to accurately assess the extent and severity of the tumor. The total volume of all metastatic lesions from the two different time points were calculated. In patients who initially presented with metastatic melanoma at TP_0_ (n = 44), we assumed the TP_-1_ status as the tumor volume of 0.1 cc 90 days prior to the baseline, considering that patients with melanoma typically undergo follow-ups involving conventional imaging, such as chest and abdominal CT scans every 3 months. To evaluate the initial volumetric change following ICB administration, gross tumor volume was delineated on imaging at the time of first response evaluation 3 or 4 cycles after ICB administration (TP_+1_) and second response evaluation 6 or 7 cycles after ICB administration (TP_+2_). The total volume was calculated for comparison with the baseline (TP_0_) sum volume.

### 2.4 Mathematical model

Patient tumor growth rate was calculated by a well-known exponential ordinary differential function (Equation 1).


Equation 1 $$\frac{dV}{dt}=\alpha V$$

where V is the total tumor volume and α is the tumor growth rate constant.

The Python curve_fit function and SciPy odient function in addition to fitting the best-fit exponential ODE curve between the data measured at TP_-1_ and TP_0_ were used to obtain an optimal fitting solution for patient’s pretreatment tumor growth rate (α). Considering that other ODE models require the determination of other parameters such as growth limit and natural death of cells, the application of the exponential model in determining tumor growth rate is relatively easy. Since our study is mainly focused on intrinsic tumor growth rate, we contemplated on using only an exponential model for the analysis. However, to compare other mathematical models, according to Murphy et al. (9), six non-exponential models were used to calculate tumor growth rate. Because these models have more than one variable, univariate analyses were performed and several mathematical models (that is. Logistic, Bertalanffy and Gompertz), which included the tumor growth limit variable, were optimized and fitted without any specific designation using the Python curve_fit function. The univariate analyses result is presented in **Supplementary Table 1**. The schematic workflow is illustrated in **Figure 1**.

To validate the calculated α by examining the correlation between α and clinical variables including changing tumor size, primary tumor site, metastasis site, and number of metastasis lesions at baseline. The correlation between α and changing tumor size and α and number of metastases was analyzed using spearman correlation, while the other variables were analyzed using the Cramer’s V correlation test. To test correlation, the primary cancer sites were classified into three categories: Cutaneous, Mucosal, and Ocular. Patients without primary site information were classified as Unknown. In addition, the study focuses on four major metastasis sites, namely the liver, lung, brain, and bone, and examines the correlation between alpha and each of these organs. For validate the interpretation of the Spearman and Cramer’s V correlation coefficient, we followed the guidelines table in **Supplementary Table 6** which is based on we used Akoglu (10) and Kim’s (11) guidelines.

### 2.5 Study outcomes

The primary study outcome was overall survival, which is defined as the time from ICB initiation to time of all-cause mortality. Secondary outcomes were (i) progression-free survival (PFS), defined as the time from treatment initiation to disease progression or death, whichever came first; (ii) PFS2, time to subsequent disease progression or death, whichever came first, after the next line of therapy; and (iii) percentage of tumor volume change at the first response evaluation (TP_+1_), defined as follows:


Equation 2 $$Tumor\;volume\;change\;\left(\%\right)=\;\frac{{\text{V}}_{\text{T}{\text{P}}_{+1}}-{\text{V}}_{\text{T}{\text{P}}_{-0}}}{{\text{V}}_{\text{T}{\text{P}}_{-0}}}\;\times 100\%$$

where $V_{TP_{+1}\;}$ is the total tumor volume at the first treatment response evaluation and $V_{TP_{-0}\;}$ is the total tumor volume at the baseline.

### 2.6 Statistical analysis

The Kaplan-Meier method was used to estimate patient’s overall survival, as well as log-rank tests to assess significant differences in survival curves, with a significance level of P ≤ 0.05. As the fitted pre-treatment tumor growth rate did not follow a normal distribution (**Supplementary Figure 1**), the cohort was divided into quartiles based on the interquartile range of α, with the upper quartile value of α = 0.076 days^-1^ identified as the threshold for the high-risk group. The study outcomes were compared between the slow-paced (≤ threshold) and fast-paced (> threshold) groups. All the other possible pairs of quartiles were compared, and the results indicated that the fastest growing tumor group (Q4) was consistently and distinctly associated with lower overall survival compared to that of Q1, Q3, or Q1-3 (**Supplementary Table 2**).

Endpoints were also be compared based on the number of metastases measured at the time pembrolizumab initiated (baseline, TP_0_), in accordance with the current definition of oligometastasis.

Sensitivity analyses were conducted by changing the tumor volume (0.1 cc to 0.01 cc or 1 cc) and interval from TP0 to TP-1 (-150 to -30 days). The analyses assessed whether the robustness of the results was dependent on the assumption that the tumor volume of patients who first presented with metastases without evidence of gross disease at TP_-1_ was 0.1 cc (number of imputed data points = 44/total number of data points = 86). We used a diameter of less than<1 cc as the minimal detectable volume, based on current CT imaging capabilities (12). P<0.05 was regarded significant. Python version 3.8.8 and log-rank test in lifelines (lifelines 0.27.1) library was used for statistical analyses.

## 3 Results

Patient and tumor characteristics are listed in **Table 1**. A total of 372, 1049, 909, and 778 gross tumor volumes of all metastatic sites were 3D contoured at TP_-1_ (86 patients), TP_0_ (86 patients), TP_+1_ (61 patients), and TP_+2_ (43 patients), respectively. The median metastatic lesion number and the total volume of all metastatic sites were 1 (range 1-61) and 0.1 cc (range 0.1-508.2 cc), 7 (range 1-73) and 28.4 cc (range 0.4-1194.8 cc), 7 (range 1-117) and 35.6 cc (range 0.5-2319.4 cc), and 7 (range 1-157) and 63.2 cc (range 0.2-4147.1 cc), at TP_-1_, TP_0_, TP_+1_, and TP_+2_, respectively. Median time interval between TP_-1_ and TP_0_ was -90 days (range -363 to -6 days).

The pretreatment tumor growth rate (α, ×10-2 days^-1^) was median 4.71 (range -0.62 to 44.1). The pretreatment tumor growth rates of the fastest (Q4) and the slowest (Q1) growing tumor group were median 9.6 (range 7.7 and 44.1) and 0.03 (range -0.62 and 0.12), respectively.

**Figure 2:**
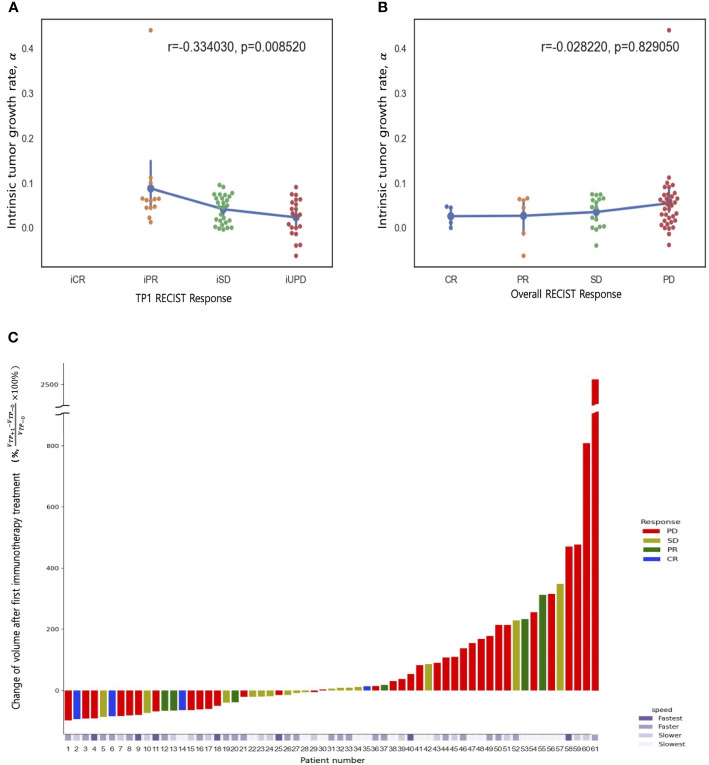
Spearman rank correlation between tumor growth rate (α) and treatment response **(A)** at first response evaluation (TP_+1_) and **(B)** the best overall response (any time from the start of ICB treatment until disease progression). **(C)** A waterfall plot of percentage changes in tumor volume compared to measurements at baseline (TP_0_) according to the best overall response category. The bottom panel represents the pretreatment tumor growth rate (α).

**Figure 3:**
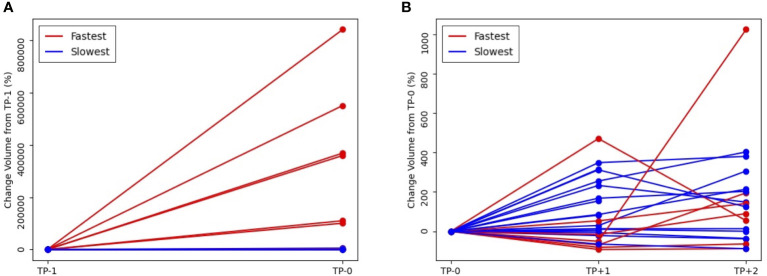
Volumetric change of tumor volume over time, according to the pretreatment tumor growth rate **(A)** from pretreatment, TP_-1_ to baseline, TP_0_ and **(B)** from TP_0_ to first response evaluation, TP_+1_ and second response evaluation, TP_+2_ (red line, fastest growing tumor group vs blue line, slowest growing tumor group).

The present study reveals a robust correlation between the rate of tumor growth and the changing tumor size, as evidenced by high Spearman rank correlation coefficient,Sp (Sp = 0.78, P = 0.03). In addition, a moderately strong association was observed between tumor growth and number of metastasis lesion (Sp = 0.52, P<.001), while metastasis at liver, lung, and brain showed moderate significance (Creamer’s V coefficient, Sc = 0.32, 0.25, and 0.31, respectively). Conversely, bone and primary tumor site exhibited weak interpretation in this regard. (**Supplementary Figure 3**, **Table 4** and **5**). The evaluation of best response as compared with tumor volume change at the first treatment response are illustrated in **Figure 2C**. A weak positive correlation was observed between the pretreatment tumor growth rate and treatment response at the first response evaluation (TP+1); however, the correlation disappeared at the final overall response evaluation (**Figures 2A–C**). **Figure 3** shows the volumetric change over time, according to the pretreatment tumor growth rate (**Figure 3A**. TP_-1_ to TP_0_, **Figure 3B**. TP_0_ to TP_+1_ and TP_+2_).

**Figure 4:**
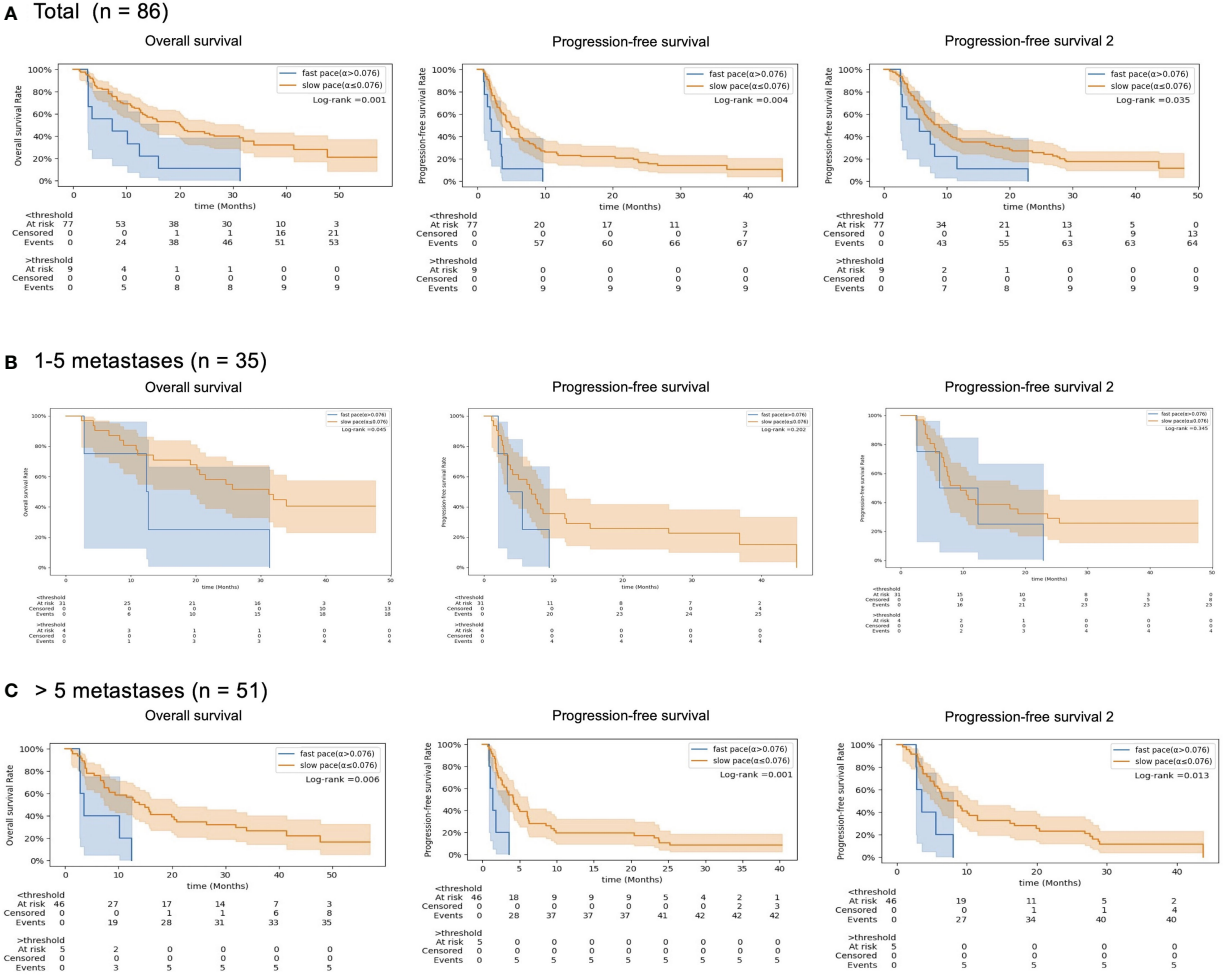
Kaplan-Meier survival curves (Overall Survival, OS), **(B)** Progression-free Survival (PFS) and **(C)** the cumulated PFS of the two first lines (PFS2.), according to the pretreatment tumor growth rate (red line, slow-paced group [α ≤ 0.075 days^-1^] vs blue line, fast-paced group (α>0.075 days^-1^)) in **(A)** total patients, **(B)** 1–5 metastases subgroup and **(C)** >5 metastases subgroup.

With a median follow up of 35 months, the 3-year OS, PFS, and PFS2 rates were 30%, 13%, and 16%, respectively. The slow-paced group had significantly higher OS (3-year, 35% vs 0%, log-rank test P =0.001), PFS (3-year, 15% vs 0%, P =0.004), and PFS2 (3-year, 16% vs 0%, P =0.035) than those of the fast-paced group (**Figure 4A**). These differences became more prominent in the subgroup of patients with >5 metastases (**Figure 4**).The median survival of each endpoint and number of metastasis subgroup are described in **Supplementary Table 3**.

**Figure 5:**
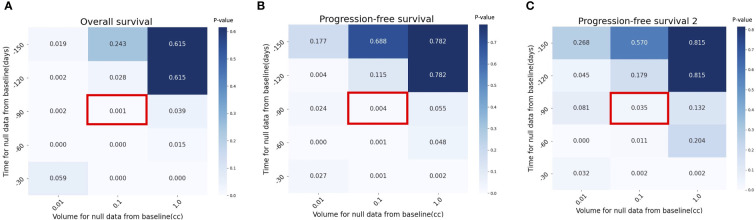
Sensitivity analysis with *p* value by different assumptions for null data at pretreatment, TP_-1_ in **(A)** Overall Survival (OS), **(B)** Progression-Free Survival (PFS) and **(C)** cumulative PFS of the two first lines (PFS2. Red box indicates the assumption used in the current study (time for null data = -90 days and volume for null data = 0.1 cc).

**Figures 5A–C** show that the results maintain their robustness in the context of different assumptions of patients without tumor volume at TP_-1_. For example, the pretreatment tumor growth rate (α) continued to be significantly correlated with overall survival under the assumption of the imputation volume of 0.01 or 1.0 cc (P=.002 and P=.039).

## 4 Discussion

We propose pretreatment tumor growth rate as a novel prognostic marker for OS, PFS, and PFS2 in metastatic patients. In the cohort of patients who had median cumulative tumor volume of 28.4 cc (range 0.4 - 1195 cc) from median 7 (range 1 - 73) metastases on baseline imaging, pretreatment growth rate was 4.71 (range -0.6 - 44.1; ×10^-2^ days^-1^). Fast-paced tumor growth rate was associated with more frequent disease progression and dismal overall survival following ICB compared to that of the slow-paced tumor growth rate. In the subgroup of patients with fewer than five metastases, as demonstrated in **Figure 4B**, there was no statistically significant difference in growth rate between subgroups, except for overall survival. While this may suggest that tumor growth rate can be disregarded in treating patients with fewer metastases, the moderately strong correlation between tumor growth rate and number of metastases indicates that the absence of statistical difference could be attributed to the small size of the data. The key finding to emphasize is that the impact of tumor growth rate on prognosis is notably significant in the subgroup of patients with more than five metastases, suggesting the potential expansion of the Oligo-Metastatic Disease (OMD) definition beyond its traditional counting-based approach. Further investigation is necessary to confirm this hypothesis. However, if tumor growth rates are slow enough to enable all targets to be treated even in patients with numerous metastatic lesions using modern radiation oncology techniques, this could indicate potential benefits of curative treatment.

Several serial imaging studies have shown that pretreatment tumor growth rate is a reliable, independent prognostic factor in early-stage non-small cell lung cancer following SABR (13, 14). In a study of 160 patients with T1-2N0M0 lung cancer, the gross tumor volume of a single lesion at initial diagnostic CT, gross tumor volume of paired lesion at SABR planning CT, and time interval (in days) between diagnostic and planning CT were used to calculate tumor growth rate as follows: ln(GTV2/GTV1)/time interval in days (14). The median tumor growth rate was 0.4 (range -2.0 - 6.1; ×10^-2^ days^-1^), which was lower than that of stage IV disease in this study (median 1.62). However, surprisingly, there was a wide range of overlap between stage I and stage IV cancer. Faster-growing tumors (≥ median), compared to slower-growing tumors (< median), were associated with increased risk of regional recurrence and distant metastasis with decreased overall survival following SABR, which implies that tumor growth rate has a substantial clinical relevance in early-stage cancers.

Recent consensus proposed a comprehensive characterization of OMD based on dynamic OMD state model (15). In this context, it might be a reasonable option to consider repeated SABRs for selected patients with recurrent OMDs, as long-term survival following repeated ablative therapies was reported (16, 17). Recently, the post-local therapy disease pace was suggested as a major consideration before offering repeat local therapy in intracranial metastases (18) and extracranial metastases (19). In 107 patients with melanoma and brain metastases treated with stereotactic radiosurgery (18), faster tumor growth (>4 cc/year) before repeat stereotactic radiosurgery was associated with a shorter time to second brain failure, increased need for salvage whole brain RT, and worse overall survival rate. In 303 patients with 1–5 extra-cranial metastases treated with SABR (19), the fast pace to second failure after first SABR was associated with subsequent widespread failure and reduced overall survival rate. Additionally, higher cumulative volume of metastases at baseline and oligo-progression/persistence type (vs. oligorecurrence) were associated with faster pace of metastases. In both studies (18, 19), tumor growth rate was calculated as the cumulative volume or numbers of new metastases divided by the time to subsequent failure. Collectively, we believe that tumor growth rate is a novel metric to differentiate actual OMDs from a transition phase to polymetastatic diseases.

This study had some limitations. Sample size, single ethnicity, and single histology suggest that the findings should be validated in independent datasets. In addition, we could not determine whether the ablation of all metastases in patients with slow tumor growth rate regardless of the number of metastatic lesions improved the overall survival rate. Due to the lack of a clinically established cut-off value for tumor growth rate, we use the definition of oligometastasis to identify the high-risk group. We have tested all possible combinations of quartile groups to avoid bias, and our results consistently demonstrate that the group with the fastest growing tumors (Q4) has lower overall survival compared to Q1, Q3, or Q1-3. However, if the threshold is changed to a lower quartile, the statistical difference between endpoints becomes difficult to identify (**Supplementary figure 2**). Further studies should determine the optimal methodological assessment of tumor growth rate and the appropriate cut-off point between oligometastasis and polymetastatic cancer.

The major strengths of this study include the manual contouring of 3,108 gross tumor volumes in patients with metastatic cancer, including polymetastastic cancers and OMDs in serial imaging and objective quantification of tumor growth rate using mathematical modeling. Mathematical modeling enables designing in-silico clinical trials that can guide the design of actual clinical trials (20–22). In this study, we began with a basic formulation of tumor growth to demonstrate its potential as an OMD biomarker. Other complexities such as metastatic locations or treatment modalities were not taken into account. They may be included in the future application but would also limit generalizability.

We have shown that pretreatment tumor growth rate in stage IV melanoma patients administered ICB can provide information on limited metastatic potential and possibility of long-term survival. In addition, it can help identify the oligometastatic patients most likely to benefit from local treatment within the capabilities of current treatment techniques. The advantage of tumor growth rate for OMD classification and in choosing appropriate treatment for the condition should be further investigated in prospective studies. Given that deep learning has shown its potential for auto-contouring normal organs (23) in the whole body and gross tumor volumes (24) in localized disease, we are currently developing deep learning-based auto-segmentation of all potential metastatic lesions as a more time-efficient alternative than manual segmentation. The advantage of deep learning-based auto-segmentation of metastases is the possibility of quickly estimating the disease burden at each time point. Thereafter, the proposed mathematical modeling can be used to calculate the pretreatment tumor growth rate using the information of disease burden change in serial imaging. Collectively, these approaches have the potential to advance the field of OMDs by improving classification and patient selection not only at the time of diagnosis of metastasis but also during follow-up.

## Data availability statement

The raw data supporting the conclusions of this article will be made available by the authors, without undue reservation.

## Ethics statement

The studies involving human participants were reviewed and approved by Severance hospital’s Institutional Review Board (4-2021-1683). Written informed consent for participation was not required for this study in accordance with the national legislation and the institutional requirements.

## Author contributions

Conceptualization: JC, WS. Formal analysis: YS, JC, WS. Resources: YS, JC, YK, SS, JK, TK, ML, RO, JSK, WS. Data curation: JC, JK, TK, JSK. Writing: YS, JC, WS. Review and editing: ML, RO, JSK. Supervision: JC, WS. Project administration: JC, WS. All authors contributed to the article and approved the submitted version.
