# Supplementary material for: Mathematical prediction with pretreatment growth rate of metastatic cancer on outcomes: implications for the characterization of oligometastatic disease
Source: Front Oncol. 2023 May 29;13:1061881. doi: 10.3389/fonc.2023.1061881 (PMC10258314; doi:10.3389/fonc.2023.1061881)

Supplementary Material

**Supplementary Table 1.** Univariate cox proportional hazard regression analysis result of 7 well-known ordinary differential equation models of tumor growth. From the exponential model, values of a were split into interquartile ranges and the first and fourth quartiles were used for the regression. For other models than the exponential, the same patient data used in regression analysis of exponential were used.

| Univariate Cox regression | | | | |
| --- | --- | --- | --- | --- |
| Variables | HR | Lower | Upper | P value |
| Exponential | | | | |
| a | 1.48 | 1.09 | 2.01 | 0.01 |
| Bertalanffy | | | | |
| a | 5.17E+9 | 12.10 | 2.21+18 | 0.03 |
| b | 163.44 | 0.04 | 6.36E+5 | 0.23 |
| Linear | | | | |
| a | 1.01 | 0.99 | 1.03 | 0.17 |
| b | 0.84 | 0.69 | 1.03 | 0.10 |
| Logistic | | | | |
| a | 0.00 | 0.00 | 0.00 | 0.03 |
| b | 3.78E+87 | 0.00 | 1.70E+187 | 0.08 |
| Mendelsohn | | | | |
| a | 2.86 | 0.38 | 21.25 | 0.31 |
| b | 2.99 | 0.18 | 49.86 | 0.45 |
| Surface | | | | |
| a | 3.31 | 0.70 | 15.75 | 0.13 |
| b | 0.00 | 0.00 | 164.20 | 0.27 |
| Gompertz | | | | |
| a | 0.00 | 0.00 | 40.81 | 0.09 |
| b | 9.10 | 0.23 | 630.91 | 0.22 |
| c | 1.41 | 0.06 | 24.16 | 0.89 |

**Supplementary Table 2.** The groups were separated by the interquartile range of α (α = Q1<Q2<Q3<Q4) and P-values were based on the log-rank test. For the conservative statistical analysis, the Bonferroni correction method (B.C.) has been adopted and the value of the table in B.C. is represented as 0.05/K, where K is the number of hypotheses. The results indicated that the fastest growing tumor group (Q4) was consistently and distinctly associated with lower survivals compared with Q1,Q3, or Q1-3.

|  | **OS** | | | PFS | | | **PFS2** | | | **B.C. (K)** |
| --- | --- | --- | --- | --- | --- | --- | --- | --- | --- | --- |
| **subgroup**  **(n)** | **Total**  **(86)** | **1-5 (35)** | **>5 (51)** | **Total**  **(86)** | **1-5 (35)** | **>5 (51)** | **Total**  **(86)** | **1-5 (35)** | **>5 (51)** | **-** |
| **Q1 vs Q2**  **Tested cases** | 0.200 | 0.732 | 0.449 | 0.432 | 0.843 | 0.187 | 0.244 | 0.747 | 0.110 | 0.008  (6) |
| **Q1 vs Q3** | 0.517 | 0.709 | 0.971 | 0.600 | 0.924 | 0.976 | 0.618 | 0.632 | 0.607 | 0.008  (6) |
| **Q1 vs Q4** | 0.009 | 0.165 | 0.037 | 0.014 | 0.351 | 0.004 | 0.039 | 0.652 | 0.025 | 0.008  (6) |
| **Q2 vs Q3** | 0.338 | 0.418 | 0.272 | 0.687 | 0.992 | 0.189 | 0.352 | 0.770 | 0.119 | 0.008  (6) |
| **Q2 vs Q4** | 0.017 | 0.106 | 0.021 | 0.013 | 0.284 | 0.026 | 0204 | 0.396 | 0.125 | 0.008  (6) |
| **Q3 vs Q4** | 0.004 | 0.052 | 0.012 | 0.016 | 0.212 | 0.004 | 0.041 | 0.286 | 0.017 | 0.008  (6) |
|  |  |  |  |  |  |  |  |  |  |  |
| **Q1 vs Q2,3,4** | 0.173 | 0.757 | 0.533 | 0.301 | 0.951 | 0.383 | 0.262 | 0.761 | 0.186 | 0.013 (4) |
| **Q2 vs Q1,3,4** | 0.499 | 0.764 | 0.021 | 0.863 | 0.756 | 0.288 | 0.434 | 0.886 | 0.165 | 0.013 (4) |
| **Q3 vs Q1,2,4** | 0.394 | 0.287 | 0.299 | 0.631 | 0.732 | 0.278 | 0.480 | 0.480 | 0.451 | 0.013 (4) |
| **Q4 vs Q1,2,3** | 0.001 | 0.045 | 0.006 | 0.004 | 0.202 | 0.001 | 0.035 | 0.345 | 0.013 | 0.013 (4) |

**Supplementary Table 3.** The median survival of each endpoint. The number of metastasis lesion were measured at the time of pembrolizumab initiation.

|  | Median survival (months) | | |
| --- | --- | --- | --- |
|  | OS | PFS1 | PFS2 |
| total | 15.87 | 4.235 | 7.865 |
| Less or equal to 5 | 25.63 | 6.6 | 9.6 |
| More than 5 | 12.7 | 3.63 | 6.47 |

**Supplementary Table 4.** Correlation between alpha and primary site

|  | Degree of freedom | Cramer’s V |
| --- | --- | --- |
| Primary site (Cutaneous, Mucosal, Ocular and Unknown) | 3 | 0.18 (Moderate) |

**Supplementary Table 5.** Correlation between alpha and metastasis site

|  | Degree of freedom | Cramer’s V |
| --- | --- | --- |
| Metastasis site | | |
| Liver included  (Yes/No) | 1 | 0.32  (Moderate) |
| Lung included  (Yes/No) | 1 | 0.25  (Weak) |
| Brain included  (Yes/No) | 1 | 0.31  (Moderate) |
| Bone included  (Yes/No) | 1 | 0.18  (Weak) |

**Supplementary Table 6. Guidelines for interpretation of the Spearman and Cramer’s V correlation coefficient.**

| **Cramer’s V correlation coefficient with effect size by degree of freedom (11)** | | | |
| --- | --- | --- | --- |
| **Degree of freedom** | **Weak** | **moderate** | **Strong** |
| **1** | **0.10** | **0.30** | **0.50** |
| **2** | **0.07** | **0.21** | **0.35** |
| **3** | **0.06** | **0.17** | **0.29** |
| **Spearman’s correlation coefficient (10)** | | | |
| **-** | **0.1<** | **0.5<** | **0.7<** |

**Supplementary Figure 1.** The distribution of the fitted alpha and quantile-quantile plot (QQplot). The median value of the data represented by a green triangle and the 3rd quartile value represented by a red triangle. The results of the Shapiro-Wilk test indicated that the distribution of alpha was non-normal (static=0.675, p-value=1.65e^-12) and QQplot show the distribution might follow heavy tailed or mixed distribution. Therefore, in this study, quartiles were employed as the data split method instead of the median.

**
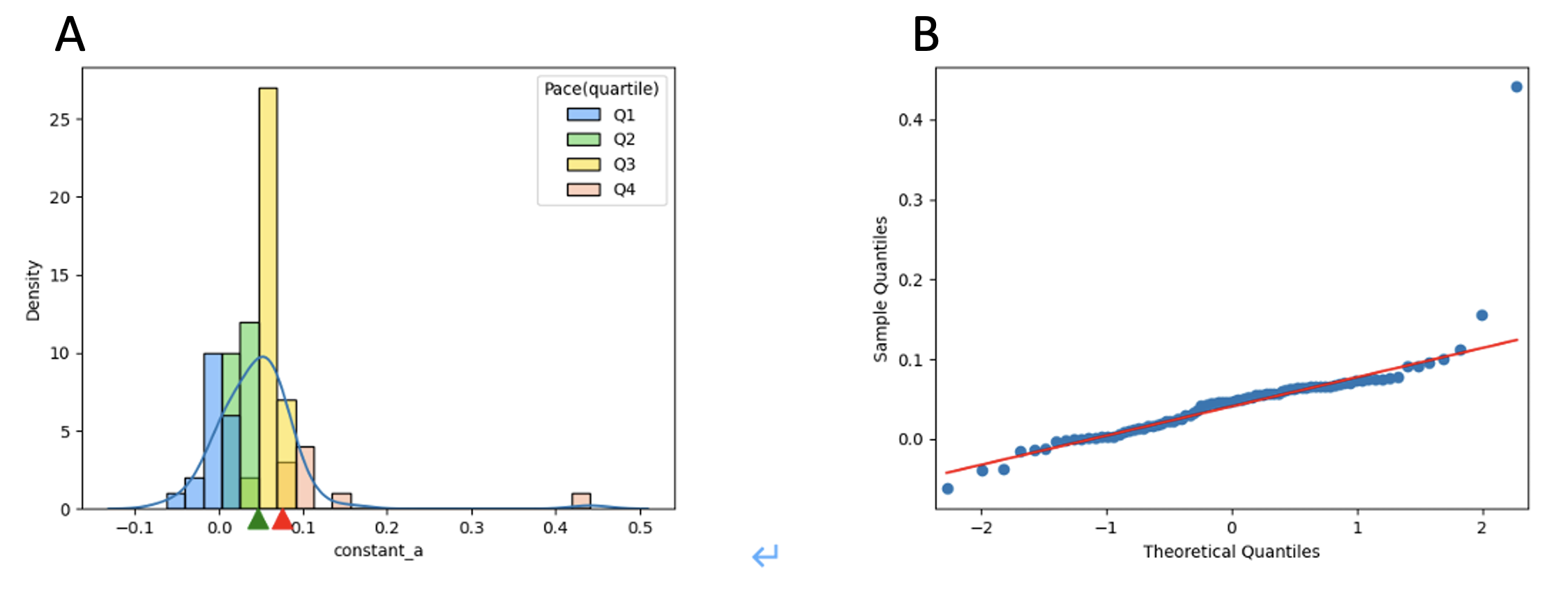
**

**Supplementary Figure 2.** Kaplan-Meier survival curves (Overall Survival, OS), (B) Progression-free Survival (PFS) and (C) the cumulated PFS of the two first lines (PFS2.), according to the pretreatment tumor growth rate (red line, slow-paced group [α ≤ lower quartile] vs blue line, fast-paced group (α>lower quartile)) in (A) total patients, (B) 1–5 metastases subgroup and (C) >5 metastases subgroup.


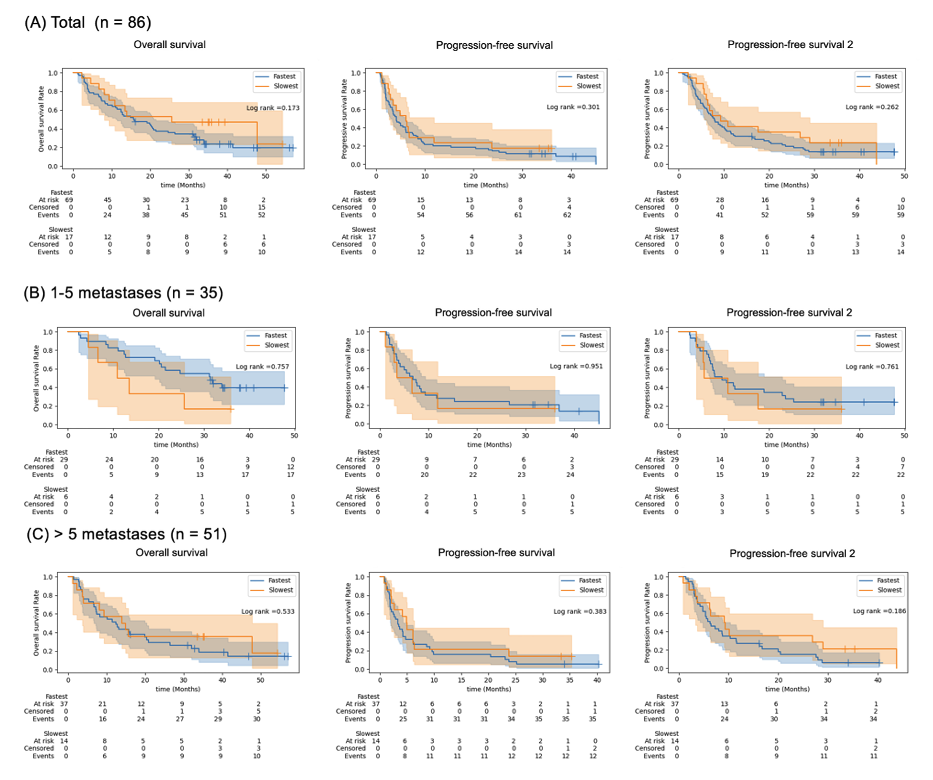


**Supplementary Figure 3.** (A) correlation between alpha and tumor size, (V_TP0_-V_TP1_) and (B) correlation between alpha and number of metastases lesion which measured at baseline.


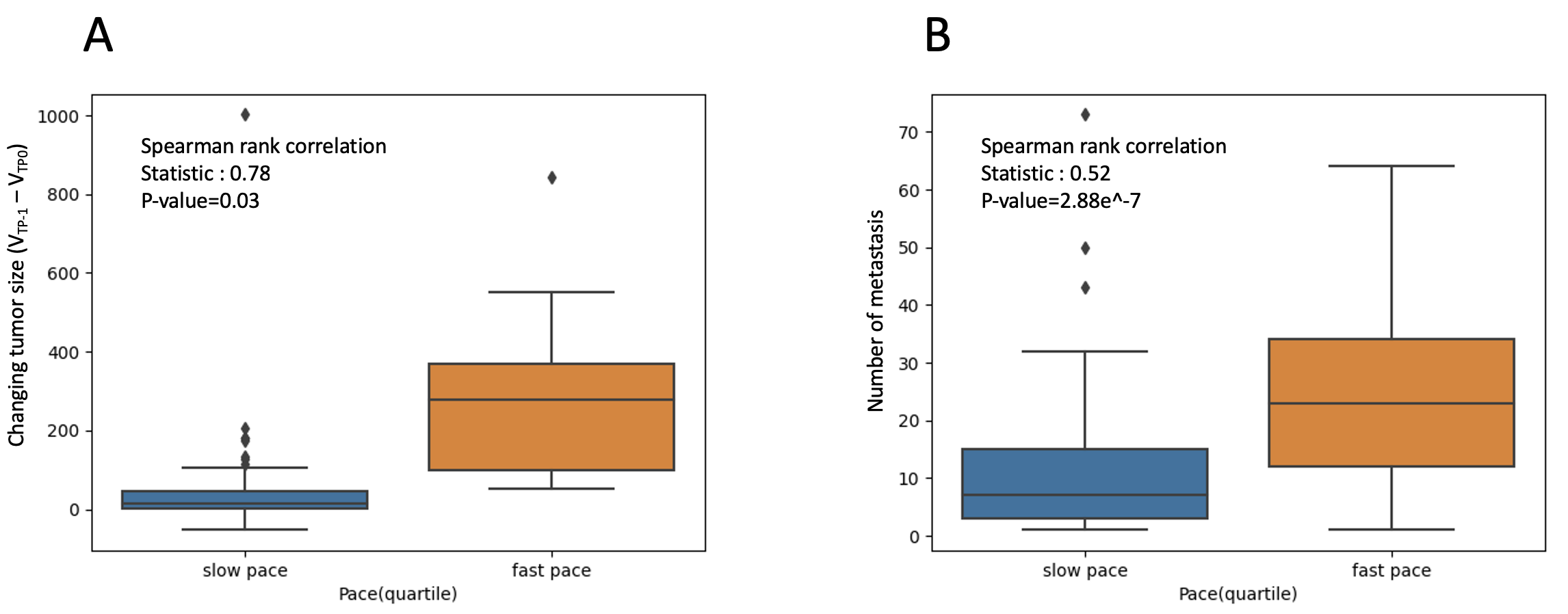

Supplement: Supplementary file 1 [file DataSheet_1.docx]
